# Supplementary material for: Chromothripsis during telomere crisis is independent of NHEJ, and consistent with a replicative origin
Source: Genome Res. 2019 May;29(5):737–49. doi: 10.1101/gr.240705.118 (PMC6499312; doi:10.1101/gr.240705.118)
Supplement: Supplemental Material [file supp_gr.240705.118_Supplemental_file_1.zip › contigs/annotated_contigs/DB56/contig.2.DB56_length_301_mean_cov_3.98671096346.docx]

**DB56_length_301_mean_cov_3.98671096346**

```
CGCTCCCTGCGAGACAGCCGCACCCGCTCCCGGGGAGAGTCATGACAACTGGGG|TC|TTGGCCTGTTCTTGCCATTTGTTGTCCAGCT
 >chr17:79370246-79370302 - E=3e-13                    >chr17:79511445-79511692 - E=1e-
CAGCTCAGTCCAACCTTCCTAAGGGACAGGTGGGTGTCCCATAGATAGGCCCAGCAGCTCCCAGTGCCCACAGCGATGCCCTCACCCTG
134
CTGCCAGAGGTGATCTGTGATGCCTGGTGTGGGGGGACGGATCTGGGCCCCCCAAGGGCGTGGCAGGATCTACATGTAGGTTGTCCACA

AGGTGTGTCACAGTAGTGGCCTTCTTCTGGAGCAGG
```
